# By the Way

**Published:** 1913-03-22

**Authors:** 


### BY THE WAY.

### ERLICH AS "HOFLIEFERANT."

The illustrious inventor of "606" being of Jewish origin, the German Emperor has been put to some pains to reward him for his discovery. The Prussian law, as a matter of fact, does not allow of Israelites being decorated or granted patents of nobility. Always ingenious, the Emperor has named him *Hoflieferant*, that is to say, Court furnisher. . . . "Honi soit qui mal y pense."—*L'Actualité médicale.*

### TEMPLE-SLEEP.

The *American Journal of Dermatology* recently published an interesting article on "temple-sleep," a curative measure much in vogue among the ancients, and consisting in spending the night on the cold damp floors of one of the sacred edifices. Naturally, the priests encouraged this means of adding to their revenues, and preached the wonderful efficacy of such therapeutic measures. Since the populace then believed that all morbid processes had their origin in supernatural interference, it was only by some supernatural agency that a cure could be found, and the priests were the proper persons to consult. It was not until the great Hippocrates came on the scene that doubts as to the utility of temple-sleep arose, partly as the result of his teaching. At a later date a revival set in under the name of "church-sleep," and as the Christian religion spread, certain churches acquired a reputation as the home of marvellous cures, so that pilgrims flocked to them from great distances. Among these was the Church of St. Andrew at Pateras, which was famous for curing stone. Mummolus, sent as ambassador by King Theudebert to the Court of Justinian, was seized with renal colic *en route*, and his death was feared. He was carried to the church to pass the night within its sacred walls, and in great suffering he sank down on the floor praying for relief. It came. About midnight, after some hours of fretful sleep, he was seized with a violent desire to micturate, and in so doing passed a calculus which St. Gregory seriously assures us was of such enormous proportions that it fell with a loud clatter into the receptacle. Thenceforth Mummolus was cured. Still more wonderful was the cure of the German Emperor Henry the Second, known as "the Saint," and who suffered from vesical stone. At this date the Italian Cloister of Monte Casino was renowned for the medical and surgical skill of its monks, and to them Henry went for treatment. Overawed by the importance of their patient, the monks determined to have no trust in earthly skill, and called upon Heaven to furnish them with an infallible means of cure, and to that end interceded, particularly with St. Benedict. The saint complied, and in person opened Henry's perineum, performed the operation usual in these days for stone, removed the obstruction, and placed it in the hands of the now happily sleeping Emperor.

